# Wheel running from a juvenile age delays onset of specific motor deficits but does not alter protein aggregate density in a mouse model of Huntington's disease

**DOI:** 10.1186/1471-2202-9-34

**Published:** 2008-04-01

**Authors:** Anton van Dellen, Patricia M Cordery, Tara L Spires, Colin Blakemore, Anthony J Hannan

**Affiliations:** 1 Department of Physiology, Anatomy and Genetics, University of Oxford, Oxford, OX1 3PT, UK; 2 MassGeneral Institute for Neurodegenerative Diseases, Massachusetts General Hospital and Harvard Medical School, Charleston, MA, USA; 3 Howard Florey Institute, University of Melbourne, VIC 3010, Australia; 4 Department of Anatomy and Cell Biology, University of Melbourne, VIC 3010, Australia

## Abstract

**Background:**

Huntington's disease (HD) is a neurodegenerative disorder predominantly affecting the cerebral cortex and striatum. Transgenic mice (R6/1 line), expressing a CAG repeat encoding an expanded polyglutamine tract in the N-terminus of the huntingtin protein, closely model HD. We have previously shown that environmental enrichment of these HD mice delays the onset of motor deficits. Furthermore, wheel running initiated in adulthood ameliorates the rear-paw clasping motor sign, but not an accelerating rotarod deficit.

**Results:**

We have now examined the effects of enhanced physical activity via wheel running, commenced at a juvenile age (4 weeks), with respect to the onset of various behavioral deficits and their neuropathological correlates in R6/1 HD mice. HD mice housed post-weaning with running wheels only, to enhance voluntary physical exercise, have delayed onset of a motor co-ordination deficit on the static horizontal rod, as well as rear-paw clasping, although the accelerating rotarod deficit remains unaffected. Both wheel running and environmental enrichment rescued HD-induced abnormal habituation of locomotor activity and exploratory behavior in the open field. We have found that neither environment enrichment nor wheel running ameliorates the shrinkage of the striatum and anterior cingulate cortex (ACC) in HD mice, nor the overall decrease in brain weight, measured at 9 months of age. At this age, the density of ubiquitinated protein aggregates in the striatum and ACC is also not significantly ameliorated by environmental enrichment or wheel running.

**Conclusion:**

These results indicate that enhanced voluntary physical activity, commenced at an early presymptomatic stage, contributes to the positive effects of environmental enrichment. However, sensory and cognitive stimulation, as well as motor stimulation not associated with running, may constitute major components of the therapeutic benefits associated with enrichment. Comparison of different environmental manipulations, performed in specific time windows, can identify critical periods for the induction of neuroprotective 'brain reserve' in animal models of HD and related neurodegenerative diseases.

## Background

Huntington's disease (HD) is a devastating autosomal dominant disorder in which neurological deterioration progresses for 10–20 years after onset, inevitably leading to death. The clinical picture is of a movement disorder, including the writhing movements known as Huntington's chorea, together with cognitive and affective impairment [1]. The pathogenic mechanism whereby the expanded CAG repeat, expressed as an extended polyglutamine tract in the huntingtin protein, induces neuronal dysfunction in the striatum and cerebral cortex is not yet understood. The normal range is 6–35 CAG repeats: HD patients have up to 250 repeats, with an inverse correlation between repeat length and age of onset of symptoms [2]. The majority of HD patients exhibit adult onset of symptoms, although juvenile-onset HD constitutes approximately 5% of cases. The availability of genetic testing means that at-risk relatives of patients can be identified prior to the onset of symptoms.

Insertion into the mouse genome of a human HD transgene, with an expanded CAG repeat, has produced several convincing disease models [3]. R6/1 mice, used in the present study, develop cognitive then motor symptoms around 3–4 months of age, becoming progressively more severe over the following months, and also model other cellular and molecular neuropathologies in HD [3-9].

We have previously demonstrated that environmental enrichment delays the onset of disease in these HD mice. Environmental enrichment, involving exposure to novel, complex objects not present in standard housing conditions, can enhance levels of sensory, cognitive and motor stimulation [10]. Environmental enrichment of the home cage delays onset of motor symptoms, judged by the appearance of the characteristic rear-paw clasping motor sign and by tests of the capacity to balance on a static horizontal rod, in R6/1 [6] and R6/2 [11] HD mice. The static horizontal rod appears to be a highly sensitive indicator of early motor onset, which was found to be dramatically delayed by environmental enrichment of these mice from a juvenile age (4 weeks) onwards [6]. Histological quantification demonstrated that environmental enrichment delays the degenerative loss of volume of cerebral cortex surrounding the striatum in R6/1 HD mice measured at 5 months, suggesting that changes in the cerebral cortex play a role in disease pathogenesis and in processes by which the disease is ameliorated [6]. Indeed, the anterior cingulate cortex (ACC) and the striatum are the first regions of the brain to undergo neurodegeneration in the R6 lines of HD mice [12], reflecting clinical neuropathology. Furthermore, early unilateral replacement of the ACC of R6/1 HD mice with healthy cortical tissue leads to amelioration of motor impairment [13].

The mechanism by which the beneficial effect of environmental enrichment occurs is unknown but the characterisation of this phenomenon might provide insight into the pathogenesis of HD. Key questions include the relative importance of mental and physical exercise, and whether there are critical periods for the initiation of environmental interventions. It has recently been demonstrated that wheel running from an adult age (10 weeks), immediately prior to motor onset, does not alter progression of the accelerating rotarod motor deficit from 15–20 weeks [14]. In the present study we compare R6/1 HD mice housed from a juvenile age (4 weeks) in either standard cages, environmentally enriched cages and cages with running wheels only, thus facilitating a specific enhancement of voluntary physical exercise. The age at which mice were exposed to running wheels was substantially earlier than the recent study [14] and a more extensive motor battery was performed (including the open field and static horizontal rod tests), allowing conclusions to be drawn about environmental interventions in gene-positive presymptomatic mice. We have differentiated the extent to which environmental enrichment and wheel running impact on behavioral deficits in HD mice and have examined cellular correlates of pathogenesis.

## Results

### Effects of wheel running on motor deficits in HD mice

**Figure 1:**
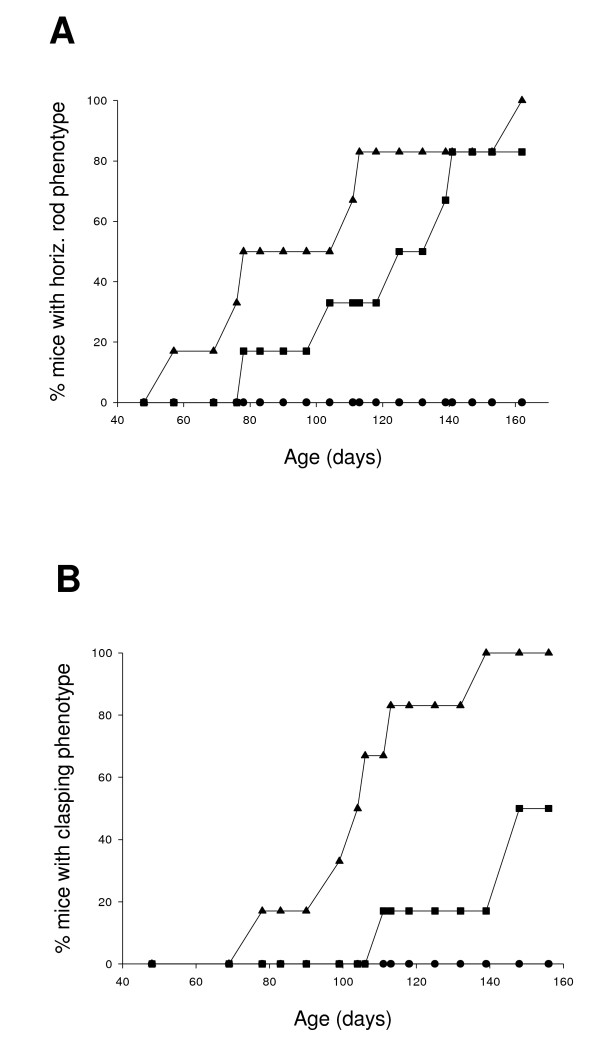
**Effects of wheel running in delaying the onset of motor deficits, measured by tests of performance on the horizontal rod and rear-paw clasping**. The cumulative percentage of mice consistently failing each test is plotted as a function of age. A) Wheel running from 4 weeks of age delays the onset of motor deficits revealed by the horizontal rod (*P *< 0.05). Wild-type mice, regardless of housing condition, always pass the horizontal rod test. B) Wheel running also delays onset of the rear-paw clasping motor deficit (*P *< 0.05). Triangles: non-enriched HD mice. Squares: wheel running HD mice. Circles: wild-type (WT) mice (enriched and non-enriched groups pooled: no failures).

By approximately 5 months of age, all non-enriched (standard-housed) HD mice fail the static horizontal rod test of motor co-ordination (Fig. 1A). Environmental enrichment dramatically delays the onset of these deficits as previously demonstrated [6]. Wheel running alone also induces a delay in onset of this motor deficit on the static horizontal rod (Fig. 1A, *P *< 0.05; Chi^2 ^Test). Furthermore, wheel running from 4 weeks also delays onset of rear-paw clasping (Fig. 1B, *P *< 0.05; Chi^2 ^Test), a reflexive motor sign in HD mice [3,6].

The accelerating rotarod test is another indicator of motor co-ordination and function. We found that environmental enrichment substantially improves performance on the rotarod for both wild-type and HD mice at 5 months of age (data not shown, enrichment: F [1, 28] = 32.93, *P *< 0.001; 2-way ANOVA), consistent with our previous observation [8].

**Figure 2:**
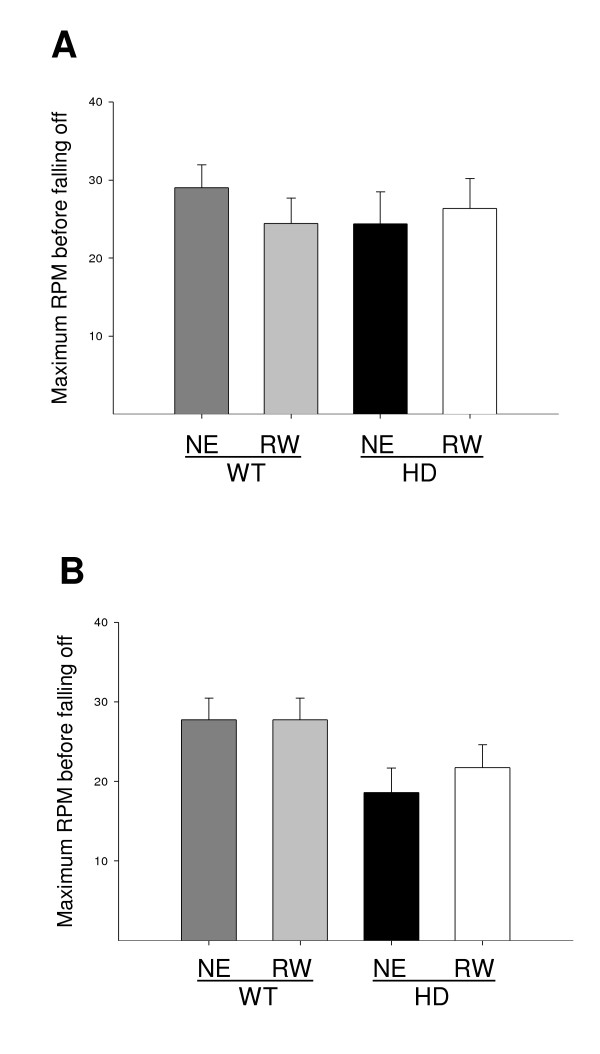
**Wheel running does not delay the onset of a motor co-ordination deficit measured on the accelerating rotarod test**. A) On the accelerating rotarod, neither the HD mutation nor wheel running (*P *= 0.68) had any affect on motor performance of HD mice at 3 months of age. B) The motor deficits which had developed in HD mice at 5 months of age were not affected by wheel running (*P *= 0.72). NE: non-enriched; RW: running wheel; WT: wild-type; HD: Huntington's disease.

At 3 months of age, neither the HD mutation nor wheel running had a significant effect on accelerating rotarod performance (Fig. 2A). Despite the fact that exercise on a running wheel is superficially similar to the testing paradigm of the accelerating rotarod, mice engaged in this form of exercise from a juvenile age did not improve performance on the rotarod in either wild-type or HD mice at 5 months of age (Fig. 2B; wheel running: F [1, 25] = 0.40, *P *= 0.72; 2-way ANOVA). At 5 months, an effect of the HD mutation on performance was observed (Fig. 2B; HD: F[1, 28] = 8.58, *P *< 0.01; 2-way ANOVA). These results are consistent with, and extend, the recent finding that wheel running starting at an adult age has no effect on progression of rotarod deficits measured from 15–20 weeks of age [14].

### Effects of environmental enrichment and wheel running on abnormal habituation of locomotor activity and exploratory behavior in the open field

**Figure 3:**
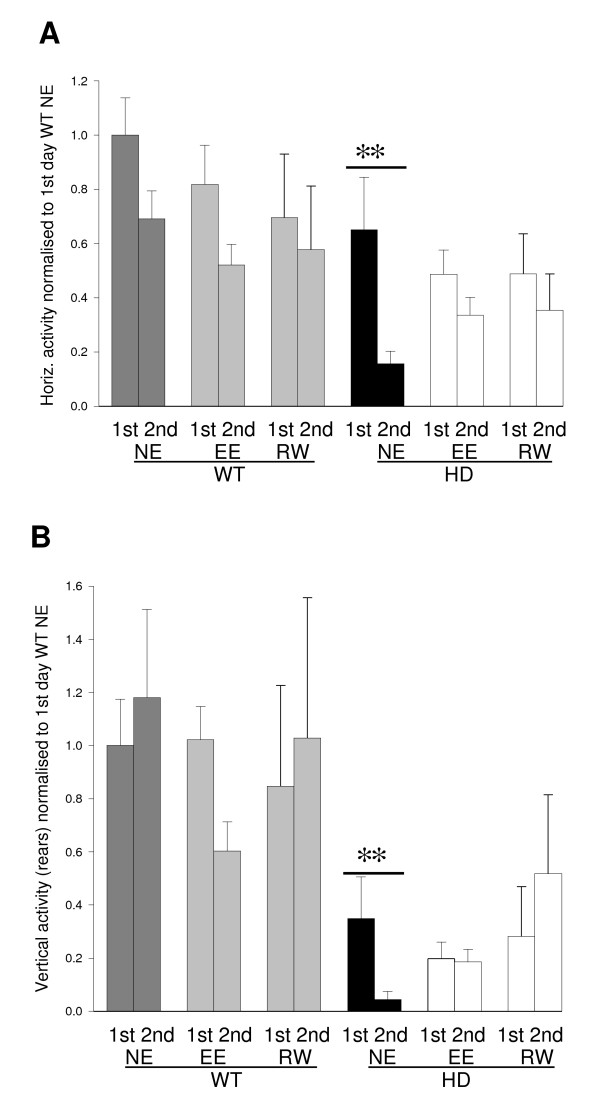
**Effects of the HD mutation and environmental manipulations on the open field test**. Spontaneous exploratory behavior was measured in terms of the horizontal activity (A) and vertical (rearing) activity (B) in the 3 min period of the open field test at 5 months of age. Interestingly, the decrease between the first and second days was highly significant for non-enriched HD mice (double asterisk: *P *< 0.001) for both the number of squares crossed (A) as well as the number of rears (B). This effect was not seen in the environmentally enriched or wheel running HD mice. This suggests that the habituation of activity seen during re-testing in HD mice is attenuated through environmental enrichment or wheel running. NE: non-enriched; EE: environmentally enriched; RW: running wheel; WT: wild-type; HD: Huntington's disease; 1^st^: first day of testing; 2^nd^: second day of testing.

In a test of exploratory behavior in the open field, carried out at 5 months of age, there was a non-significant trend for HD mice to be less active than wild-type littermates (Fig. 3A) (HD: F [1, 38] = 3.71, *P *< 0.10, 2-way ANOVA). Although in some groups of mice exploratory activity was reduced on the second day of testing (2nd blocks in Fig. 3A), this reduction was highly significant only for non-enriched HD mice (HD: F[1,38] = 22.29, *P *< 0.001; 2-way ANOVA). This demonstrates that the abnormal habituation of locomotor activity in HD mice was rescued by both environmental enrichment and wheel running.

Episodes of rearing (vertical orientation balancing on rear limbs) were also recorded in the open field test. The general paucity of rearing in HD animals compared with wild-type mice was particularly pronounced on the initial day of testing (1st blocks in Fig. 3B: F [1, 38] = 63.22, *P *< 0.001; 2-way ANOVA). As with square-crossing, there was a dramatic decrease in activity on the second day of testing (2nd blocks in Fig. 3B) in non-enriched HD mice (F[1,38] = 38.25, *P *< 0.001; 2-way ANOVA). Thus environmental enrichment and wheel running both rescued the abnormal habituation of rearing behavior in HD mice.

### Environmental enrichment and wheel running do not rescue HD-induced decreases in body weight or brain weight at 9 months of age

As expected, expression of the HD transgene resulted in a significant decrease in body weight by 9 months (HD: F [1, 28] = 104.76, *P *< 0.001; 2-way ANOVA). Both environmental enrichment (Table 1) and wheel running (Table 2) were incapable of abating the weight loss induced by the transgene (Table 1, environmental enrichment: F [1, 28] = 28.31, *P *= 0.27; Table 2, wheel running: F [1, 21] = 2.76, *P *= 0.11; 2-way ANOVA).

**Table 1:** Environmental enrichment did not ameliorate the effects of the HD mutation on body and brain weight at 9 months of age. The HD transgene affected both body weight (*P *< 0.001) and brain weight (*P *< 0.001), relative to WT littermates. NE: non-enriched (standard housed); EE: environmentally enriched.

	WT		HD	
	
	EE	NE	EE	NE
Body weight (grams)	36.74 ± 1.75	40.02 ± 2.24	20.73 ± 0.56	21.24 ± 0.98
Brain weight (grams)	0.44 ± 0.013	0.45 ± 0.008	0.34 ± 0.013	0.36 ± 0.012

**Table 2:** Wheel running did not ameliorate the effects of the HD mutation on body and brain weight at 9 months of age. The HD transgene affected both body weight (*P *< 0.001) and brain weight (*P *< 0.001) at 9 months of age, relative to WT littermates. NE: non-enriched (standard housed); RW: running-wheel housed.

	WT		HD	
	
	RW	NE	RW	NE
Body weight (grams)	39.70 ± 3.01	32.36 ± 2.57	22.38 ± 1.05	22.54 ± 0.75
Brain weight (grams)	0.54 ± 0.015	0.53 ± 0.008	0.42 ± 0.011	0.42 ± 0.007

The presence of the HD transgene also significantly reduced overall brain weight at 9 months (HD: F [1, 36] = 16.01, *P *< 0.001; 2-way ANOVA). Neither environmental enrichment (Table 1) nor wheel running (Table 2) mitigated this effect (environmental enrichment: F [1, 36] = 0.12, *P *= 0.73; wheel running: F [1, 12] = 0.11, *P *= 0.75; 2-way ANOVA).

### Environmental enrichment and wheel running do not rescue degenerative shrinkage of the striatum and anterior cingulate cortex in HD mice at 9 months of age

**Figure 4:**
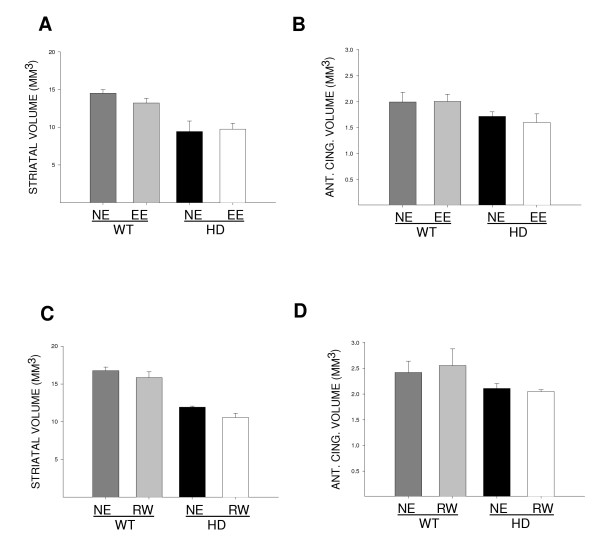
**Volumetric measurement of neurodegeneration in the striatum and anterior cingulate cortex (ACC)**. The volumes of the striatum and ACC were calculated for non-enriched (NE), environmentally enriched (EE) and wheel running (RW) groups of wild-type (WT) and HD mice at 9 months of age for the striatum (A and C) and ACC (B and D). The presence of the transgene reduced all these brain volumes, reaching statistical significance for both striatum (*P *< 0.001) and anterior cingulate cortex (*P *< 0.05). No effect was seen on the striatum or anterior cingulate cortex by either environmental enrichment or wheel running.

We analysed serial coronal sections to measure the volume of the striatum and the anterior cingulate cortex (ACC) in environmentally enriched (Fig. 4A,B) and wheel running (Fig. 4C,D) cohorts. Expression of the HD transgene reduced the volumes of both the striatum and the ACC (striatum: F [1, 12] = 39.74, *P *< 0.001; ACC: F [1, 12] = 5.24, *P *< 0.05; 2-way ANOVA).

By 9 months of age, environmental enrichment had no significant effect on striatal atrophy (Fig. 4A) or ACC atrophy (Fig. 4B). Similarly, neither striatal (Fig. 4C) nor ACC (Fig. 4D) atrophy was significantly affected by wheel running.

### The density of protein aggregates in the striatum and ACC at 9 months of age is not significantly altered by environmental enrichment or wheel running

Neuronal inclusions, or aggregates, are formed by aggregation of huntingtin (htt) protein fragments containing the expanded polyglutamine tract, as well as ubiquitin and other proteins, and are a distinctive feature of HD brains at post mortem [15]. These aggregates are also seen in transgenic HD mice, where they were first described [16], and appear before the onset of explicit disease symptoms. However, it is not clear whether they play a causative role in HD pathogenesis. Aggregates are never seen in wild-type mice at any age. We previously found that there is no significant effect of environmental enrichment on the overall density of ubiquitinated protein aggregates in the striatum of R6/1 HD mice [6].

**Figure 5:**
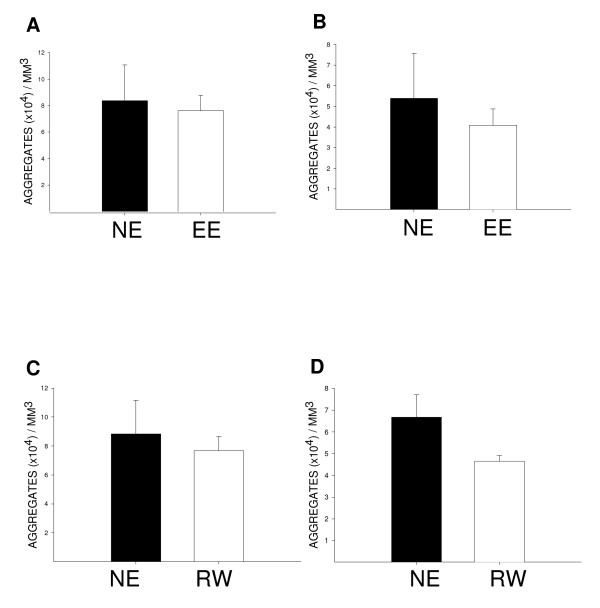
**The density of ubiquitin-positive protein aggregates in the striatum and anterior cingulate cortex (ACC) of HD mice**. Stereological analysis of ubiquitin immunoreactive protein aggregates was performed for the striatum (A) and ACC (B) of environmentally enriched (EE) HD mice at 9 months of age. The same analysis was performed for the striatum (C) and ACC (D) of wheel running (RW) HD mice. Neither environmental enrichment nor wheel running affect the density of aggregates in the striatum (A and C) or the anterior cingulate cortex (B and D) of HD mice at 9 months of age. As expected, these aggregates were never observed in wild-type mice.

We calculated the densities of ubiquitin-positive aggregates in brains from 9-month-old HD transgenic mice. The two areas examined were the striatum (Fig. 5A,C) and the ACC (Fig. 5B,D), key areas of inclusion pathology in HD [11]. In keeping with findings for environmental enrichment at 5 months of age [6], neither paradigm significantly alters the density of aggregates at 9 months of age. This was true for both the striatum (Fig. 5A, environmental enrichment: *P *= 0.81; Fig. 5C, wheel running: *P *= 0.67; Student's *t*-test) and the ACC (Fig. 5B, environmental enrichment: *P *= 0.60; Fig. 5D, wheel running: *P *= 0.16; Student's *t*-test), although there was a non-significant trend for wheel running to decrease aggregate density in the anterior cingulate cortex (Fig. 5D).

## Discussion

Although HD has previously been considered to be a disease that is the epitome of genetic determinism, it is increasingly recognised that environmental factors can modulate disease onset and progression [10]. Environmental enrichment is known to have a number of beneficial effects, including delaying onset and slowing progression of motor deficits in multiple transgenic mouse models of HD [6,8,11,17], although the relative contribution of sensory, cognitive and motor stimulation to these therapeutic effects has not been described.

The present study demonstrates that enhanced physical exercise via wheel running alone is capable of delaying the onset of specific motor deficits in HD. Both environmental enrichment [6] and wheel running delayed the onset of certain signs of disease, as measured by the horizontal rod and clasping tests of motor function. However, the beneficial effect of wheel running on disease onset did not extend to rescuing performance on the accelerating rotarod, a test of motor co-ordination. Of particular interest was the fact that in the present study wheel running was commenced at a juvenile age, much earlier than a recent study in which wheel running was initiated in adulthood and progression of rotarod deficits at 15–20 weeks was found to be unaffected by running, despite an observed delay in rear-paw clasping [14]. The present results also demonstrated for the first time that wheel running can delay the onset of motor coordination deficits on the static horizontal rod, but has no effect on accelerating rotarod deficits.

The rotarod results were somewhat surprising, given the superficial similarity of activity involved in both wheel running and balancing on the accelerating rotarod. However, the precise patterns of posture and coordination are substantially different in wheel running and balancing on the rotarod. Running voluntarily for long periods each day over several months inside a freely-moving concave wheel provides no advantage in learning to maintain balance on the convex outer surface of the accelerating motorised rotarod, for either wild-type or HD mice.

In the present study we also show that HD-induced abnormal habituation of locomotor and rearing activity in response to repeated testing in the open field is rescued by both environmental enrichment and wheel running. The habituation seen in standard-housed HD mice, which was not observed in their wild-type littermates housed in the same standard conditions, may reflect altered memory of their previous exposure to the open field. Therefore, environmental enrichment and wheel running from a juvenile age may be rescuing a cognitive deficit in this instance. This is consistent with our recent demonstration that wheel running initiated during adulthood can rescue a spatial working memory deficit in HD mice [14]. Furthermore, we have recently demonstrated that environmental enrichment of HD mice delays onset of a spatial cognitive deficit on the Barnes circular maze [18].

Various mechanisms might account for the effects of environmental stimulation in delaying the onset of neurological signs in HD mice. We have previously shown that environmental enrichment does not alter the density of ubiquitin-positive protein aggregates in the striatum of R6/1 HD mice at 5 months of age [6]. In the present study, neither environmental enrichment nor wheel running had any significant effect on the density of ubiquitinated aggregates in the striatum or anterior cingulate cortex at 9 months of age. This would imply that these forms of environmental stimulation do not exert their beneficial effects through modulating numbers of protein aggregates.

In the present study we also examined the volume of the striatum and anterior cingulate cortex at 9 months of age, and found that neither environmental enrichment nor wheel running had a significant effect on HD-induced shrinkage of these brain regions. The striatal data is consistent with our previous study where we found that environmental enrichment did not affect striatal shrinkage in R6/1 HD mice at 5 months of age [6]. However, in these same brains at 5 months of age, we found that prior enrichment significantly rescued the HD-induced loss of 'peristriatal cerebral volume', a measure which predominantly consisted of the neocortex surrounding the striatum [6]. In the present study, we examined a far more specific cortical region, the anterior cingulate cortex, at 9 months of age, and saw no significant volumetric effect of environmental enrichment. This could be because we examined a different brain area, or because we made the measurements at a later age. Our past and present data suggests that environmental enrichment delays, but does not prevent, onset of specific molecular, cellular and behavioral deficits, and therefore investigations at later ages may reveal less dramatic effects.

The expression of specific neurotransmitter receptors and signalling molecules is decreased in the cortex and striatum of R6/1 and R6/2 HD mice prior to cell loss [4,19-21]. Environmental stimulation might ameliorate excitotoxicity via reduced transcriptional dysregulation of various neurotransmitter receptors and synaptic signal transduction pathways. For example, downregulation of cannabinoid CB1 receptors, which is seen in HD patients at post-mortem [22], occurs in R6/1 HD mice and is rescued by prior enrichment [23], presumably leading to changes in neurotransmission at synapses expressing these receptors. Brain-derived neurotrophic factor (BDNF) is a neurotrophin whose deficit has been implicated in HD pathogenesis [8,14,24-29]. We have shown that environmental enrichment can rescue the deficit of mature BDNF protein in the striatum of R6/1 HD mice [8]. However we have recently shown that wheel running does not rescue deficits of BDNF expression in the R6/1 HD brain [14]

There is now substantial evidence for disrupted synaptic function, including plasticity, in the corticostriatal system from a range of different HD models [30]. Abnormal synaptic plasticity, including long-term potentiation (LTP), in hippocampal slices have been described for R6/1 [31], R6/2 [32] and other HD mouse models [33,34], and perirhinal cortical synaptic plasticity is also disrupted in R6/1 mice [35]. Early deficits in neocortical plasticity in R6/1 mice have also been described [36,37]. Environmental enrichment in rats increases the strength of specific cortical synapses, influences LTP, and increases the binding of glutamate to AMPA receptors [38]. In wild-type mice, wheel running has been shown to enhance hippocampal LTP [39]. Environmental enrichment, including enhanced physical exercise on running wheels, may therefore ameliorate the defects in the HD mice by directly overcoming deficiencies of synaptic function, including synaptic plasticity.

Furthermore, there is evidence that another form of cellular plasticity, adult neurogenesis, is disrupted in the hippocampus of R6/1 [40,41] and R6/2 [42,43] HD mice, and that environmental enrichment may ameliorate this hippocampal neurogenesis deficit [44]. The demonstration that wheel running can ameliorate deficits of spatial memory in HD mice [14], which are know to be dependent on the hippocampus, is of interest, particularly as wheel running is known to enhance hippocampal neurogenesis in wild-type mice [45].

These results show that environmental enrichment has a somewhat greater beneficial effect that wheel running in mitigating the effects of HD in transgenic mice, even when wheel running was started at a juvenile age. In particular, environmental enrichment delayed onset of a motor coordination deficit on the accelerating rotarod [8], whereas in the present study wheel running did not significantly affect this specific motor deficit. This suggests the possibility that sensory stimulation, mental exercise and physical activity could all be employed in attempts to harness environmental enrichment for the benefit of human sufferers. Strategies of occupational therapy based on the principles of environmental enrichment may be beneficial for gene-positive presymptomatic individuals and patients with HD, even those with fairly advanced disease [46,47], for which there is currently no accepted treatment. A study of Venezualan kindreds provides strong evidence for a role of environmental factors in modifying age of disease onset in HD [48], although the nature of these environmental modifiers is unknown. It was calculated that 60% of the variability seen in age of onset of HD after accounting for the effect of CAG repeat length is due to environmental factors [48]. This is supported by the fact that monozygotic twins with confirmed identical CAG repeat lengths can present with different symptomatology [49].

These findings may have broader implications for other brain disorders. Mental and physical engagement with a stimulating environment is thought to have beneficial effects in delaying dementia in Alzheimer's disease [50]. Recent studies have demonstrated beneficial effects of environmental enrichment and wheel running in transgenic mouse models of Alzheimer's disease [51-54], as well as various other brain disorders involving the adult cortex and striatum [10,55,56]. Such environmental manipulations may thus allow us to model 'brain reserve', or more specifically 'cognitive reserve' in the context of dementia, and the underlying neuroprotective mechanisms [10]. We have also recently demonstrated differential effects of environmental enrichment and wheel running on onset and progression in a transgenic mouse model of amyotrophic lateral sclerosis (ALS), highlighting important differences in the quality and quantity of motor stimulation associated with these two environmental manipulations (Stam *et al*., submitted).

## Conclusion

We have demonstrated that both environmental enrichment and wheel running can rescue abnormal locomotor habituation of HD mice in the open field. We have also provided evidence that wheel running from a juvenile age can delay onset of some, but not all, motor deficits in HD mice. Our results suggest that a combination of enhanced mental and physical activity may be optimal in delaying the onset of Huntington's disease. Environmental enrichment and wheel running do not, however, alter the density of protein aggregates in the anterior cingulate cortex or striatum at 9 months of age, suggesting that these large intracellular aggregates may not be directly involved in pathogenesis. However, early stages of pathological protein aggregation and protein-protein interactions remain promising targets in the search for HD therapeutics [57]. Finally, environmental manipulations provide powerful tools for elucidating the molecular and cellular mechanisms of pathogenesis, with the identification of potential novel molecular targets for therapeutic intervention [58], which may have implications for the treatment of HD and other neurodegenerative diseases.

## Methods

### Animals

Male R6/1 mice [3] (Jackson Laboratories, USA), after back-crossing onto the CBA background for greater than 10 generations, were mated with female CBA mice to produce experimental cohorts. The offspring were randomised into environmentally enriched, wheel running and non-enriched (standard housed) groups. The enrichment was through either exposure to novel objects (environmental enrichment) or access to running wheels (both described below). At 1 month of age, the mice were tail-tipped for genotyping [3], and a microchip (Labtrac, Uckfield, UK) was inserted subcutaneously, under Halothane anaesthesia, for identification purposes. The genotype coding was broken only at the end of the experiments. As expected, approximately 50% of mice in each litter were positive for the HD transgene and the remainder served as wild-type controls. All animal work was conducted in accordance with the United Kingdom Animals (Scientific Procedures) Act of 1986 and was approved under a Home Office project license.

### Environmental enrichment and wheel running housing conditions

All mice, including those in standard housing conditions, were group-housed to control for any effects of social interaction, with 4–6 animals in standard rodent cages (measuring 44 × 28 × 12.5 cm). Environmental enrichment consisted of novel objects placed in the home cages from 4 weeks of age [6,8]. These were changed every two days without additional handling of the mice. Enrichment objects included small cardboard boxes, either enclosed or open at one end, 15 ml and 50 ml plastic conical tubes (Becton Dickinson, New Jersey, USA), cylindrical cardboard tunnels approximately 3 cm in diameter, and folded sheets of paper approximately 10 × 10 cm. No edible or toxic substances were added to the cage. Control littermate mice receiving routine care (non-enriched) had normal feed, bedding and social contact. For wheel running cohorts of mice, two small metal running-wheels (8 cm diameter) were placed in each cage from 4 weeks of age, without any other form of environmental enrichment. The cages were monitored regularly and this confirmed that the wheels were frequently used by the mice, with either one or two mice observed to run on a single wheel.

### Behavioral analysis

For all behavioral experiments, mice were tested by an observer who did not know the genotype of the mice.

#### Open field

Spontaneous motor activity was assessed for each mouse at 5 months of age. The open field was a grey PVC enclosed arena, 50 × 30 cm, divided into 10 cm squares. Each experimental mouse was placed into a corner square facing that corner, and the number of squares entered (whole body) was counted over a 3 min period. Rearing of the body (both front paws off the ground, but not as part of grooming), and grooming episodes (number of bouts and total duration) were also noted. The number of squares crossed by wild-type non-enriched mice on the first day of testing was normalised to 1 to facilitate comparison between groups of environmentally enriched, wheel running mice and non-enriched mice, of both genotypes. The open field test was repeated on a second day in order to quantify habituation of activity.

#### Accelerating Rotarod

The mice were placed on a Rotarod (Ugo Basile model 7650, Sandown Scientific, Hampton, UK) at an initial rate of 3.5 revolutions per minute (rpm) with an acceleration of 20 rpm/min to a maximum of 40 rpm [8]. The central cylinder was 3 cm in diameter and had 2 mm ridges along its longitudinal axis. Two flanges, 30 cm in diameter, were set at each end of the cylinder, at a separation of 6 cm, and the mouse was placed on the rotating central cylinder between the flanges. Mice were familiarised with the task prior to measurements being taken.

#### Static horizontal rod test

The mouse was placed on the end of a horizontal, wooden rod, 21 mm in diameter, facing towards the end of the rod, which was suspended approximately 30 cm above an open box containing soft bedding [6]. The rod was firmly secured at the other end. In this situation, mice attempt to turn around, and move to the fixed end. We measured the time taken for the mouse to turn 180 degrees. The mouse was judged to have failed this task if it took more than 100 seconds or fell off the rod. Disease onset was defined as the first of an unbroken sequence of weekly trials on which the mouse consistently failed, with an allowed exception of a single subsequent successful test.

#### Rear-paw clasping

The clasping of the hind limbs together, which is observed in parallel to onset of other motor signs, was analysed as previously described [6].

### Immunohistochemistry and quantitative analysis of tissue sections

Mice were terminally anaesthetised with halothane (Halothane-RM: Rhone Merieux, Harlow, UK) followed by intraperitoneal pentobarbitone (Euthatal: Rhone Merieux; 200 mg/kg), and perfused with phosphate-buffered saline (Sigma, St Louis, USA), followed by 4% paraformaldehyde (TAAB, Aldermaston, UK) in phosphate buffer (PB) (BDH, Poole, UK). The brains were then dissected free and weighed (Precisa 125A, Precisa, Switzerland). All procedures were conducted as authorised by the relevant project and personal licenses issued by the UK Home Office.

The paraformaldehyde-fixed brains were submerged for 24 h in a solution of 30% (w/v) sucrose (BDH) in PB. Coronal sections of the forebrain were cut at 40 μm covering the entire anteroposterior extent of the striatum. Immunohistochemistry was performed as described [59] on alternate sections with a 1:3,000 anti-ubiquitin (rabbit polyclonal: Dako, Glostrup, Denmark). Slides were coded independent of the microchip number of the mouse and examined by an observer who was blinded to the genotype or housing conditions of the mice. Ubiquitin immunoreactive cells were counted in sample sites throughout the striatum and cortex by means of an automated stereological analysis system (Stereoinvestigator, MicroBrightField, Colchester, USA). In every sixth section, 15 sample sites were randomly allocated by software within the defined boundary of the striatum on each side. At each site, ubiquitin-positive neuronal aggregates were counted via an optical dissector (50 μm × 50 μm) with a 3D counting frame height of 30 μm. The presence of aggregates also independently identified mice carrying the HD transgene, agreeing in every case with the result of PCR genotyping. For each section containing the striatum, the boundaries of the striatum were recorded and digitised. The anterior cingulate cortex was defined as cerebral cortex superior to the striatum, medial to a line drawn vertically from the most superior point of the striatum. We calculated the respective volumes of these two defined brain regions and combined the values from both sides of the brain. In combination with cell counts, these volumes were used for cell density calculations.

### Statistics

A Chi^2 ^Test was conducted on the horizontal rod testing and clasping data. A 2-way Analysis of Variance (ANOVA) was conducted on the open field and rotarod behavioral testing data and brain volume measurements. A student's t-test was conducted on the counts of ubiquitin-positive aggregates.

## Abbreviations

ACC, anterior cingulate cortex; BDNF, brain-derived neurotrophic factor; EE, environmental enrichment; HD, Huntington's disease; htt, huntingtin; NE, non-enriched; RW, running wheel.

## Authors' contributions

AVD generated the mouse cohorts, performed behavioral testing and co-wrote the manuscript. PMC performed the immunohistochemistry and stereological analysis. TLS contributed to the behavioral experiments. CB provided co-supervision and helped to draft the manuscript. AJH conceived of the study, participated in its design and coordination, contributed to behavioral experiments and co-wrote the manuscript.
